# Potentiation Physiologically Proven by Professor Jaeger

**Published:** 1892-06

**Authors:** G. Jaeger

**Affiliations:** Stuttgart, Germany


### POTENTIATION PHYSIOLOGICALLY PROVEN.

BY PROF. DR. G. JAEGER, STUTTGART, GERMANY.

[Translated from the Leipzig *Allgem. Homœop. Zeitung*, Vol. 124, No. 11, by B. Fincke, M. D.]

### I. PREAMBLE.

As the readers know, I have accepted the invitation of the editors to take up again this subject, which I have already treated in three publications, and to report thereon to the readers of this journal.

Originally I intended to divide the work into parts, and to publish every part as soon as done. But, as a general rule, in a greater work it happens that the point from which the author starts is not always the point from which the reader takes the matter in the easiest way. This is especially the case when both parties do not stand on the same ground, and this is here the case.

As the readers of my first publication, *Neural Analyse der Homœopathischen Verdünnungen*, Leipzig, 1881 (also contained in *Entdeckung der Seele*, third edition, Vol. 2), know, a simple *practical need* caused me to occupy myself with the subject of potentiation. I had discovered a physiological test, *Neural Analysis*, and it was desirable to determine how far its testing power reached. In this way I came unawares upon the *homœopathic attenuations* which hitherto had never troubled me, and which I, in company with all schoolmen, had taken to be "nothings." Now, it was extraordinarily instructive for me (1) That, indeed, as Hahnemann quite correctly recognized, the attenuation is not a weakening, but an augmentation of its efficaciousness; (2) that neural analysis is a test method of almost incredible applicability.

Supposing the adherents and representatives of Homœopathy who were scoffed at and despised by the old school on account of the attenuating process would be interested in an exact method to prove scientifically the doctrine of potentiation, I have published the investigation just as I and three of my former pupils have made and found it.

The homœopathic literature, it is true, accepted the publication gratefully, and saluted me as ally in the fight against the allopathic school. In this way I was next pushed into a distinct direction, viz.: the *polemical toward the outside.* I threw the facts found by me in diverse small publications at the heads of the adversaries of Homœopathy, and some of these thrusts, indeed, came home beautifully, but I soon saw that the adherents of Homœopathy did not fancy a lively, aggressive warfare against allopathy, and so I gave the matter the cold shoulder.

The reverse, however, is this: On the part of Homœopathy, its adherents forgot altogether, in their joy at having found an aggressive, or, at least, a defensive weapon against the enemy *outwardly* to draw the consequence of my joy *intra muros.* Two things had to be considered in this respect:

(*a.*) The facts published by me spoke with the strongest possible logic, viz., that of number, for the administration of *high*

*potencies* and against the use of *low* potencies. Hence, my publication found an undivided acknowledgment only by the " high potentialists," whilst the adherents of the lower potencies acted as all the schoolmen did ; instead of allowing themselves to be instructed, and of drawing the practical deductions from my paper, they tried to circumvent the matter, and they remained just as they were before.

(*b.*) My paper showed that there is a method of numerical computation of the height of potentiation, respectively, that the, by me invented, neural analysis offers the means of demonstrating a method by which, being *in practice* placed before a homœopathic vial, one could ascertain whether its contents had gone approximately through that potentiation which had been prescribed and inscribed upon it. Also, this consequence has *not* been drawn, and so our ways separated—*i. e., mine* and those of the homœopaths—and with this I have arrived at the pith of what I want to say.

After I had convinced myself—especially when writing the essay, *Neural Analyse der Homœopathischen Verdünnungen*—of the practical significance of neural analysis, *I advanced to the practical development of the application of the method upon my own territory, Hygiène,* and especially in contrast to the representatives of Homœopathy who did *not* follow the suggestion on my part, and did *not cultivate practically their own* territory. I do not say this to express a reproach, but only to establish an incontestable fact.

With me there was this difference: my practical activity was in the department of *Hygiene*, and not in that of *Therapy*, and the difference between these two departments must be clearly understood if the difference of standpoint between me and the representatives of Homœopathy is to be comprehended.

*Therapy*, the *healing art*, up to the day when the Reichstag gave liberty to *internal medicine* is neither *de jure* nor, till today, *de facto* a *free art*, but a *scholastically* and *bureaucratically*, rather *ligated* art, which is teeming with dogmas, school opinions, prejudices, discretions, views, old-fashioned rules, practices, ordinances, laws, prohibitions, so that one cannot make one free step without running the risk of treading on the corns of another—yea, even of getting into conflict with the authorities. I know this not only from observation as an outsider, but it has come from my own experience on this ground, when I stepped on it with my *Anthropin*, and the same seems to repeat itself with the *Autoisopathy** which was recommended by me. Therefore, two things are clear: (1) For the outsider the pleasure of interfering is very much diminished after burning one's fingers once. (2) For the unfortunate who stands entirely upon this hot ground, and naturally burns his fingers the easier, of course, the desire and possibility of introducing a change anywhere is about equal to zero, and the result is everything remains *in statu quo*.

Here a difference between *Homœopathy* and *Allopathy* appears which in the department of Homœopathy causes a much greater difficulty of changing anything in the historical condition than upon allopathic ground, and he who tries to move anything from its place in the department of Homœopathy must be perfectly clear on this point.

Allopathy has two "*enfants terribles*" which did not allow it any rest, but even drove it directly into a systematic longing for innovation; the *poison* and the *knife*. The fear of both, physician and patient against both, the evident mischief which they both do, allows no conservatism to come up. Thus the very latest time, on the territory of Allopathy, has produced a feverish hastening after the new, and the spectacle of Kochine has had the greatest effect in the allopathic world, where all, physician and patient, lost their heads completely over a "novelty" (which it was not, after all).

In comparison with this, *Homœopathy* lives in *profoundest peace*, physician and patient likewise; for even if the latter is not cured, if the homœopathic physician has no better success than his allopathic colleague, he and his clients are satisfied there is no poison and no knife; the torture of selecting the remedy is *per se* only a small one and only felt more by one

---

* Autoisopathy is a new word of Jaeger's own coining.

part. If now, besides, the homœopathic physician sees how his allopathic colleague in feverish unrest driven by poison and knife, plunges from one mire into another, he feels himself still more confirmed in his conservatism; he elevates this standpoint to a virtuous principle and is dripping almost all over with contentment and devotion, so that it is almost impossible to make anywhere any improvement.

Let us compare with this the *Hygiene*. This is and remains a *free art*, and it is to be hoped that also the *hygienic chairs* of the university erected two decades ago cannot change anything about it. There is no such thing as hygienic scholasticism; yea, because the medical scholastics allowed this territory to lie fallow till about twenty years ago, and since, of course, the scholastico-bureaucratically-led public till then naturally never cared for Hygiene, he who moves upon this territory is a *free-man*, and his only guide is *practical success*. Here, indeed, also are certain haunting dogmas, old-fashioned customs and abuses, manners, and the like, and thorns are not wanting, but one is not opposed by a closed, scholastically organized, compact power, as the school-medicine is on the territory of Therapy, and one moves on a ground where not examination, title, and rank, but solely the success, decides.

This, now, was the ground upon which my Neural Analysis was developed. The description in my work "*Neural Analyse der Homœopathischen Verdünnungen*" was a method which is very much suited to put a *scientific* question and to answer it, but it is unsuitable in *practice*.

First, the apparatus used at that time, the chronoscope of Hipp, was impractical because clumsy, and I replaced it by the pocket chronoscope, which already in its first trial furnished the greatest advantages in comparison with Hipp's chronoscope, and now in its second form fulfills all requirements of practice.

With this instrument I entered upon the problems of Hygiene, not only in the department of habiliment, but also in that of the articles of food and luxury, and other objects of use, and now I look upon a decennial practice—*i. e.*, an activity which comprises in itself a continual co-operation on my part as Neuroanalytician with men of practice, experts in their own fields who do not accept an I for an U. In this decennial practice I have developed my method, have adapted it to the practical needs and nature of the subjects, have found the conditions under which favorable results are obtained; in short, I am through it all, and the practical men who worked with me have submitted to my Neural Analysis, though not without struggle, because it always hit the mark, and never left me in the lurch. The matter is finished on this ground; there is nothing more to try, but only to practice according to clear directions.

Now, the diversity of the objects is to be mentioned shortly.

In my neuro-analytical practice the test of the purity of the object is concerned, and the determination of the degree of fineness—*e. g.*, with spirituous liquids, tobacco, cigars, preserves, differences of quality in metals, papers, wrappings, etc. Objects, therefore, are in question, which differ from homœopathic medicines, especially in that, indeed, also, fineness is concerned, but relatively masses which cannot be annihilated by a breath. Since the publication on Neural Analysis, I have, with few exceptions, not done anything for a long time, especially nothing in a practical direction, because I am neither a homœopathic physician nor an apothecary.

This was the state of things when the request of the editors arrived. I have responded immediately, and worked since the middle of November, so that two series of investigations are finished and ready for print. One is already in the printing-house for some time.

The next result was, as could not be expected otherwise, a confirmation of the same experience as ten years ago: THE ATTENUATION IS REALLY A POTENTIATION, and the higher the potency the higher the animating power of matter. Furthermore, the finished investigations furnish an essential extension in five directions:

1. Formerly the proving proceeded only by inhalation. Now a potency-series of one substance is submitted to deglutition, and the nerve-time is proved during seven to fourteen minutes continually.

2. Whilst the first time only four most possibly different substances (Gold, Kitchen-salt, Aconite, and Thuja) had been proved, this time seventeen nearly related substances have been thoroughly proved.

3. Whilst the first time the lower potencies were shortly disposed of, and the higher were only examined in greater intervals, I laid the point of gravity this time especially upon the lower potencies and upon the determination of the point of *indifference.* This has the advantage that we here move upon the field of poisonous action known to the other side, and that thus the method is controlled by the experiences of toxicology.

4. The very interesting and also practically important result was gained that there are substances which do not bear potentiation, which, in a certain height, decompose, as, similarly, there are substances which do not bear too high a degree of heat.

5. It was found that we can convince ourselves also without chronoscope of the correctness of the principle of potentiation by observation of other physiological phenomena, nay, even that we can recognize, to a certain amount, the degree of potentiation.

The reader will admit that these are remarkable extensions, and comprehend that I wanted to begin my publication with the communication of these results, and thus lead the reader immediately in *mediam rem.* The manuscript was already in the printing-house, when I thought otherwise, and for this reason:

In my present provings by inhalation I have kept within the *scientific* method, as at the first time, viz., I have examined *every potency by itself separately*, because I told myself that it would be very difficult to examine *different potencies* of the *same substance* simultaneously or in immediate succession. On the other hand, I convinced myself that it is always feasible to examine and compare the *same potency* of *different* substances in immediate succession because they influence each other very little.

When, in the Society of the Homœopathic Physicians of this city I communicated briefly my results, they placed me immediately before the *practical* side of the question, asking:

"Can you, at our next meeting, if we place before you a few vials with alcohol and different potencies of the same substance, the contents of which you do not know, recognize by your method the different potencies?"

I declared at once, that, after supper in my inn, where people drink and smoke and several persons are present who by their close attention influence the examiner suggestively, the conditions for neural analysis are as unfavorable as possible; that to this must be added that different potencies of the same substance even under favorable conditions cannot well be compared. Nevertheless, I would be ready to try, since at least it must interest myself whether anything or what could be gained by it.

Before making this experiment the problem was proposed to me from another side. I received four vials, of which one contained alcohol and the others three different potencies of the same substance without indicating what it was. An experiment which I made at home, under the same conditions as usual, miscarried, as I supposed beforehand. Several potencies of the same substance cannot be compared at the same time. The later experiment made in the Society gave the same result. At last a record of measurements of my son, Dr. M. Jaeger, homœopathic physician in Hall, made at my instigation, arrived. He had, just as in other cases, measured *different* potencies of the *same substance* (30, 100, 200, 1,000, and a two per cent. solution of Arsenic-salt) successively—*i. e.*, with five minutes intervals—without knowing what he measured, and found each time long series of numbers (twenty to twenty-two decades), but at the same time committed the great fault that before measuring he made an inhalation for one whole minute, so that he must have missed the most important variations.

If, now, in this manner, as I generally do, a rest-number is compared with a medicine-number formed from all twenty decades, the result was perfectly confused. But, after separating the twenty decades into five mean-values of four decades each and determining the maximal differences of the decade numbers in rest and medicine-action, it was found that from the three first measurements the correct result could be extracted, and that only the two last were unfit for the diagnosis, because saturation with the substance, blunting, and fatigue had totally destroyed the necessary disposition for measurement, which appears from the first glance at the rest-numbers.

Now, I looked at the single numbers which I had obtained in my experiment in the Society: every four decade-numbers measured during rest and inhalation of medicine were each time combined to a mean-number. Whilst now the mean-numbers gave a *false* diagnosis, the examination of the *single* numbers showed that with the two first vials, the result would have been right if I had measured four further decades and ignored the four first ones. In a nerve system impregnated with victuals, beer, smoke, and human exhalations, the action can hardly be as rapid as when the experimenter is free from such influences and alone. The third measurement also, which was not quite incorrect, would, if continued, perhaps have given a correct result, and only the fourth was and remained useless when looking at the single numbers.

From these experiments I next drew the following conclusions:

It is easy by neural analysis to prove the *scientific* side of potentiation and to obtain remarkably exact results, but *in praxi* difficulties arise in the application of the method. These partly lie in the nature of the *object*. With high potencies finesses are concerned which by far exceed what I had to determine in my hygienic practice, and which, therefore, make other precautionary measures necessary which I use in my present practice. The other difficulties lie in the *subject*. It is ten to one that every practitioner makes the same claim of the method as has been made twice to me, and as my son made it to himself in his own experiments: "Can I discriminate with the method *different potencies* of the *same* substance and discriminate promptly?" This is a question which, without thorough investigation—*i. e.*, without a whole series of varying measurements—cannot be decided—*i. e.*, I believe that it may be done, if not too much is asked at once, but it must even be determined what must be done and what not, and what is to be thought of the numbers.

I remind the reader only of the following: Hahnemann has given exact instructions what, in the use of homœopathic medicine, is to be done and what not: (1.) To take the medicine on an empty stomach. This also is required for neural analysis. (2.) To do it in a quiet disposition—the same in neural analysis. (3.) He forbids the use of numerous substances—*e. g.*, coffee, tea, perfumery, Camphor. If Hahnemann, as I doubt not, is right (of Camphor I know it for certain), then also in neural analytical provings of medicine the same prohibitions must be observed, for the influences which cancel the therapeutical action of the attenuated medicine substances weaken or annihilate their *physiological* action; therefore it can, of course, not accomplish what neural analysis is to show in numbers, if nothing is going on in the body.

Now I return to what I said at first: I have taken hold of the work at the same end as in 1881. The reader, ten to one, wants to take hold of the *practical* end, and this cannot be done, because the handle to it has not yet been made.

I am, consequently, placed in a difficult alternative.

If I publish the two series of provings, I run the risk that the matter will be taken hold of, especially by those readers whom I desire, viz.: by those who oppose experiment to experiment, and not rhetorical exercise to experiment in the wrong way, and that, therefore, it will be discredited.

The other alternative is that I delay the publication till the practical handle for the matter is made and the method adapted to the practice.

As a *matter of fact*, the latter is decidedly the right way, but it means a delay of publication for at least half a year.

"Why?" Very simply. I work now—to-day is the first of March—since three and a half months, in this matter to the neglect of my own work. Whilst I intended this winter to carry out only one series of measurements, I now have already done two, and shall I now do a *third?* For that I have neither time nor desire, especially at the approach of spring, when I am tired of the four walls. This is, however, only one reason; the other is the following:

As I, ten years ago, during the scientific proving, had desired not to be left *alone*, so I feel exactly as to the *practical* proving. I have expressed this desire already in my letter to the editors (published in Nos. 25 and 46 in the 123d volume of this journal), but now it has grown to a *conditio sina qua non. If in this affair I do not find any assistants in the ranks of the homœopathic physicians or apothecaries, I let them alone;* for, for myself, this side of the subject has no interest, because I am neither a homœopathic physician nor apothecary.

I am willing to continue alone of caring for the *scientific* side of the subject. Assistants are also here welcome, but they are not necessary. If, however, the gentlemen of practice have not so much interest in the solution of the *practical* question that I can find assistance, then there is also no reason I should do this thing alone and publish it. No rooster will crow over it, and to work for this my time is too precious.

Thus far the matter is very simple, but in the meantime I have been placed in a constrained position. The table of curves for the first series of measurements has been cut already a month ago. The manuscript for this first series which likewise went to press several weeks ago has been retained by me, as soon as I saw how matters stood. Instead of returning the paper the printer sends me—whilst I am writing—the proof-sheets of the first part, hence the paper is already in type.

Without sacrifice this cannot be changed, and so may the first part make its way into the public such as it is. It is to be hoped that the insight into what can be done if there is a will, will have the result that I shall find what I need.

But first I wish to say what I do not want.

It has come to my knowledge that already after the announcement of my view in No. 25, vol. 123, a critic pointed his pen for a round with me which goes against all and every rule of "comment." The rule not only in the fencing-room but also on the field of science must be—*equal arms*. My weapons are the *experiment* and the *number*. If anybody opposes me with *other* weapons, with *rhetoric* and *phraseology*, I must decline. I give no satisfaction with unequal arms. What I want are assistants, not *critics*. This demand must be made also by the reader, or else if in a journal always on one side stands the *author* and on the other side the *critic*, the reader finds himself in the fatal position of the donkey in the fable who starved between two bundles of hay.

[TO BE CONTINUED.]

